# Geospatial Relationships between Awareness and Utilization of Community Exercise Resources and Physical Activity Levels in Older Adults

**DOI:** 10.1155/2014/302690

**Published:** 2014-10-16

**Authors:** Christopher J. Dondzila, Ann M. Swartz, Kevin G. Keenan, Amy E. Harley, Razia Azen, Scott J. Strath

**Affiliations:** ^1^Department of Health, Exercise, and Sport Science, The Citadel, Deas Hall, Room 113, 171 Moultrie Street, Charleston, SC 29409, USA; ^2^Department of Kinesiology, The University of Wisconsin-Milwaukee, Enderis Hall, 2400 East Hartford, Milwaukee, WI 53201, USA; ^3^Center for Aging and Translational Research, The University of Wisconsin-Milwaukee, NWQ-B, Room 1427, Milwaukee, WI, USA; ^4^Zilber School of Public Health, The University of Wisconsin-Milwaukee, Zilber School of Public Health Building, Room 409, 1240 North 10th Street, Milwaukee, WI 53205, USA; ^5^Department of Educational Psychology, The University of Wisconsin-Milwaukee, Enderis Hall, Room 769, 2400 East Hartford, Milwaukee, WI 53201, USA

## Abstract

*Introduction*. It is unclear if community-based fitness resources (CBFR) translate to heightened activity levels within neighboring areas. The purpose of this study was to determine whether awareness and utilization of fitness resources and physical activity differed depending on residential distance from CBFR. *Methods*. Four hundred and seventeen older adults (72.9 ± 7.7 years) were randomly recruited from three spatial tiers (≤1.6, >1.6 to ≤3.2, and >3.2 to 8.0 km) surrounding seven senior centers, which housed CBFR. Participants completed questionnaires on health history, CBFR, and physical activity, gathering data on CBFR awareness, utilization, and barriers, overall levels, and predictors to engagement in moderate to vigorous physical activity (MVPA). *Results*. Across spatial tiers, there were no differences in positive awareness rates of CBFR or CBFR utilization. Engagement in MVPA differed across spatial tiers (*P* < 0.001), with the >3.2 to 8.0 km radius having the highest mean energy expenditure. Across all sites, age and income level (*P* < 0.05) were significant predictors of low and high amounts of MVPA, respectively, and current health status and lack of interest represented barriers to CBFR utilization (*P* < 0.05). *Conclusion*. Closer proximity to CBFR did not impact awareness or utilization rates and had an inverse relationship with physical activity.

## 1. Introduction

Older adults (65+ years) are among the most rapidly growing segments in the United States population, and projections predict this trend to continue into the future [1, 2]. Despite modern advancements in medicine and technology, there are continual health concerns in the older adult population. The prevalence of chronic conditions, such as diabetes, osteoporosis, hypertension, hyperlipidemia, obesity, and cancer, remains high, having a detrimental effect on an older adult's overall health and quality of life and placing excessive economic strain on our nation's health care system [3, 4]. Accordingly, there is an increased emphasis on exploring the effectiveness of preventative efforts to ameliorate the burden of such adverse health outcomes in older adults.

Regular physical activity and exercise have long been promoted as a means to treat and prevent a multitude of health conditions [5], yet the number of older adults who are regularly active is staggeringly low. Based on objective physical activity assessments, it is estimated that only 3.5–10% of older adults are meeting physical activity recommendations [6, 7]. Furthermore, the amount of physical activity performed across the older adult years steadily decreases as sedentary behaviors begin to dominate everyday life [8, 9].

There is a complex interaction of factors that influence habitual physical activity engagement. A key barrier to physical activity for the older adult population is access to resources that promote regular physical activity and exercise [10–12]. Community-based fitness resources (CBFR) can provide older adults a wealth of opportunities to promote increases in physical activity levels, such as removing/minimizing barriers to physical activity, including the availability, supervision, and instruction on use of exercise equipment, and developing a supportive network of peers. Such factors have been shown to be critical in influencing physical activity levels in older adults [13]. Proximity to CBFR is likely to be important, as it further reduces a potential transportation barrier [10–13] and may also result in a greater awareness of programming opportunities and their associated benefits.

Senior centers offer an excellent conduit in which to promote CBFR and could serve as an organizational mediator to physical activity behavior in older adults. To date, it remains unclear whether proximity to senior centers with CBFR has an impact on awareness and utilization of resources and ultimately overall physical activity levels of older adults. Thus, the purpose of the current study was to assess awareness and utilization of CBFR, based on residential spatial tiers of increasing distance from said resources. It was hypothesized that individuals living in closer proximity to CBFR would have greater awareness, utilization rate, and overall higher physical activity levels, compared to those residing further away from CBFR.

## 2. Methods

### 2.1. Study Design

This cross-sectional study involved gathering a series of information regarding awareness and utilization of CBFR and current physical activity levels, based on proximity to the facilities. Participation consisted of the completion of a series of questionnaires mailed to participants, which included a health history questionnaire, a community-based resources questionnaire, and the CHAMPS physical activity questionnaire. In addition to the questionnaires, a cover letter was enclosed to orient the participant on completing the forms, as well as a preaddressed, stamped envelope for the questionnaires to be returned to the investigative team.

The surrounding areas of seven local senior centers with CBFR throughout a large metropolitan area were included in the current study. Extensive calling lists of those aged ≥60 years were compiled to recruit potential participants. These lists were designated to include all older adults residing within 5 miles of targeted senior centers, obtained through marketing companies. Calling lists were then segmented by geographic information systems software into those who resided ≤1.6, >1.6 to ≤3.2, and >3.2 to 8.0 km from targeted senior centers. Within the stratified calling lists, a random sample of potential participants was contacted via telephone to inquire if they would be interested in participating in this study. Upon receiving verbal consent to participate, as approved by the University's Institutional Review Board, all documents were sent out in the mail. All data collection was conducted within a single season, thus reducing the confounding of seasonality on responses.

### 2.2. Participants

Inclusion criteria for participating in the study consisted of being between 60 and 90 years and willingness to complete and return all questionnaires. By nature of the study design, all participants contacted were previously stratified to be residing within 8.0 km of a targeted senior center.

### 2.3. Study Measures

#### 2.3.1. Community-Based Fitness Resource Questionnaire

An 11-point questionnaire was developed by the investigators to amass descriptive data pertaining to CBFR awareness, utilization, transportation, and barriers to utilization.

*Community-Based Fitness Resource Awareness*. To assess awareness of CBFR, participants checked a box either “yes” or “no” to the following question: “Are you aware of any exercise/fitness programs or classes at your local senior center?” 

*Community-Based Fitness Resource Utilization*. To assess utilization of CBFR, participants checked a box either “yes” or “no” to the following question: “Do you currently attend or participate in any of the exercise/fitness programs or classes at your local senior center?”

*Barriers to Community-Based Fitness Resource Utilization*. Additionally, participants were given the opportunity to identify what barriers pertaining to CBFR use were applicable to them from the following question: “What barriers prevent you from attending and participating in any exercise/fitness programs or classes at your local senior center more often/if at all?” A list of common barriers was provided, including knowledge of services, time, transportation, work/other commitments, health, lack of interest, and distance from resources, prompting participants to check a box adjacent to each applicable barrier that contributed to limiting their engagement. There was no limit to how many barriers could be marked as influencing CBFR utilization.

#### 2.3.2. Physical Activity Assessment

The CHAMPS physical activity questionnaire was used to collect information on physical activity engagement, targeting frequency (days/week) and weekly duration spent engaging in various exercise behaviors, everyday activities, and leisure-time activities common to older adults. For the current study, the outcome measurement from the CHAMPS questionnaire was weekly caloric expenditure in moderate to vigorous intensity activities, using adapted MET values for older adults [14]. Calculating energy expenditure from the CHAMPS questionnaire requires calculating weekly duration engaged in each activity, which has been shown to have acceptable measures of reliability, with *r* values ranging from 0.67 to 0.76 [14, 15]. The CHAMPS questionnaire has also been shown to appropriately demarcate varying physical activity levels with a level of precision similar to more intensive physical activity interviewing assessments (*F*_2,246_ = 20.85, *P* < 0.001), providing evidence for the CHAMPS questionnaire to be a valid physical activity assessment tool [14].

### 2.4. Data and Statistical Analysis

Descriptive statistics are expressed as mean ± standard deviation. Chi-square tests were performed to examine if awareness and utilization rates of CBFR differed across spatial tiers. Results were calculated as the overall percentage of those who responded “yes” to the inquiries on awareness and utilization over the total sample. Kruskal-Wallis tests were performed to examine if engagement in moderate to vigorous physical activity (MVPA) differed across spatial tiers. Multinomial logistic regression analyses were performed to identify which mediators to physical activity (age, gender, income, car ownership, and CBFR utilization) were significant predictors to overall activity levels, represented by caloric expenditure. The dependent physical activity categories included sedentary (0 kcals/wk; referent category), low levels of physical activity (>0–6710 kcals/wk), and high levels of physical activity (>6710 kcals/wk). The cut point used to delineate low and high physical activity levels was based on the median energy expenditure values among all nonsedentary (>0 kcals/wk) participants in the current sample. Binary logistic regression analyses were performed to identify which barriers significantly inhibited CBFR utilization, including knowledge of services, time, transportation, work/other commitments, health, lack of interest, and distance from resources. All statistical analyses were performed utilizing SPSS 19.0 for Windows (Chicago, IL).

## 3. Results

### 3.1. Participant Characteristics

**Figure 1:**
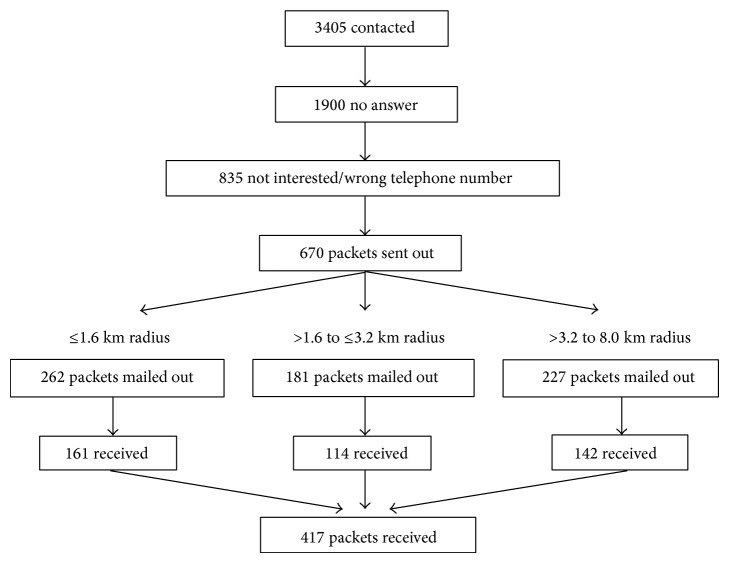
Participant flow diagram.

A total of 3405 participants were contacted for participation in this study.  Figure 1 depicts the recruitment flow, leading to the final sample of 417 older adults. Of the final sample, 161 were included in the ≤1.6 km radius group, 114 in the >1.6 to ≤3.2 km radius, and 142 in the >3.2 to 8.0 km radius. The successful return rates of complete questionnaires for the aforementioned spatial tiers were 61.5%, 63.0%, and 62.6%, respectively.

Participant demographics are listed in Table 1. There was an even distribution of female (*n* = 208) and male (*n* = 206) respondents, and participants were primarily Caucasian, had at least a high school education, and owned a car. No clear trend was discernable between education and income levels with car ownership across spatial tiers, although little variation was evident among these variables to allow such a distinction to be made. Body mass index (BMI) for all participants averaged just below the threshold (30 kg/m^2^) to define obesity [16].

### 3.2. Community-Based Fitness Resource Awareness and Utilization

**Figure 2:**
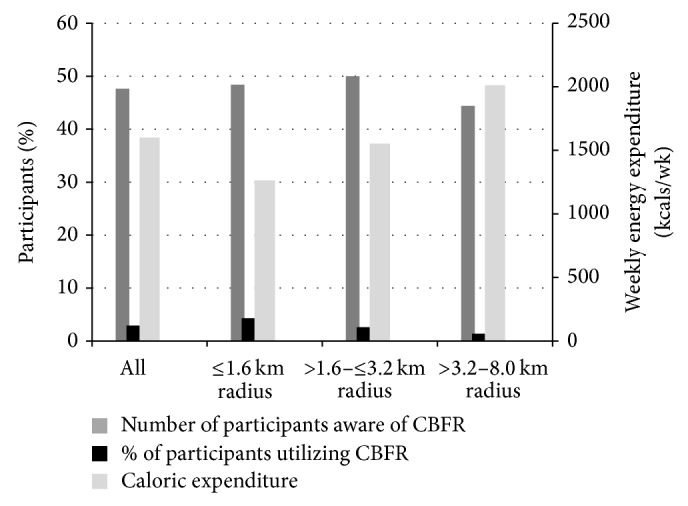
Awareness and utilization of community-based fitness resources (CBFR) compared to weekly energy expenditure (mean ± SE).

The responses for awareness and utilization of CBFR are reported in Figure 2. Among all respondents in the ≤1.6 km, >1.6 to ≤3.2 km, and the >3.2 to 8.0 km radii, 48.4%, 50.0%, and 44.4% were aware of CBFR, respectively. Respondents' overall utilization rates of CBFR, however, were extremely low (2.9%), with no differences across the spatial tiers (*χ* ^2^ = 2.37, df = 2, *P* = 0.306). Among those residing in the ≤1.6 km, >1.6 to ≤3.2 km, and the >3.2 to 8.0 km radii, only 4.3%, 2.6%, and 1.4% of participants responded positively to utilizing CBFR, thus exhibiting a weak trend of decreased utilization with increasing distance from CBFR.

### 3.3. Barriers to Community-Based Fitness Resources

Among the barriers listed that had a negative influence on CBFR utilization, lack of interest in CBFR was the most frequently cited barrier (51.6% of participants), followed by time (18.2%), work (16.1%), health (14.1%), transportation (9.1%), and distance (2.9%). Including all participants across all spatial tiers, only health (*β* = 1.408, *P* = 0.004) and lack of interest (*β* = −2.302, *P* = 0.002) were significant predictors of individuals not utilizing CBFR. Specific to spatial tiers, the only significant barriers were transportation (*β* = 5.47, *P* = 0.002) in the >1.6 to ≤3.2 km radius and health (*β* = 2.27, *P* < 0.05) in the >3.2 to 8.0 km radius.

### 3.4. Physical Activity Engagement

The average energy expenditure in MVPA for all participants across all sites was 1601 ± 2293 kcals/wk (*n* = 378), represented in Figure 2. Engagement in MVPA differed across spatial tiers (*χ* ^2^ = 15.74, df = 2, *P* = 0.000), with mean caloric expenditures rising in conjunction with increasing distance from CBFR: from 1263 ± 2177 kcals/wk (*n* = 146) to 1555 ± 1793 kcals/wk (*n* = 101) to 2013 ± 2680 kcals/wk (*n* = 131), respectively. Overall, 27.8% reported an energy expenditure of 0 kcals/wk (*n* = 105), 29.1% from >0 to 999 kcals/wk (*n* = 110), 8.7% from 1000 to 1499 kcals/wk (*n* = 33), 8.5% from 1500 to 1999 kcals/wk (*n* = 32), 5.8% from 2000 to 2499 kcals/wk (*n* = 22), and 20.6% >2500 kcals/wk (*n* = 78). Including participants from all spatial tiers, the multinomial regression model accounted for 16.7% of variability in MVPA values, with age and income being significant predictors of low (*β* = −0.04, *P* < 0.05) and high (*β* = 0.92, *P* < 0.05) levels of physical activity, respectively. Specific to spatial tiers, age was a significant predictor of low levels of physical activity within the ≤1.6 km radius (*β* = −0.062, *P* < 0.05) and >2–5 mile radius (*β* = −0.07, *P* < 0.05). No other independent variables were significant predictors of low or high levels of physical activity.

## 4. Discussion

National data suggest only a small percentage of older adults are active enough to receive the health benefits of physical activity, raising the susceptibility to poor overall health with increasing age. One approach to promoting physical activity and exercise specific to older adults is through local senior centers, providing an environment conducive to support physical activity and exercise by way of exercise equipment/rooms and supervised fitness classes. Such CBFR aim to reduce the influence of barriers that negatively impact regular physical activity, including lack of access to facilities, guidance, and social support. Still, other factors remain potentially unresolved by CBFR that contribute to their utilization (or lack thereof). Mainly, the influence of the availability of transportation and lack of time constraints remain unaffected and are heavily governed by one's residence distance from such resources. However, it is unclear how awareness and utilization of CBFR are thus impacted by distance from centers promoting and providing resources for active lifestyles. The main findings of this study show that, among spatial tiers of increasing distance surrounding CBFR, there were no statistical differences in awareness or utilization of CBFR. Moreover, despite approximately one-half of participants being currently aware of CBFR, utilization rates were less than 5% among those surveyed. Additionally, respondents reported greater physical activity levels the further one resided from CBFR.

An estimated 25% of older adults report utilizing senior centers [17], providing a promising setting for physical activity promotion efforts. Despite this, the results of the current study suggest that CBFR have a negligible impact on physical activity. There was a substantial decline in the number of individuals who used CBFR, relative to those who were aware of the resources (only 3% utilized CBFR, out of approximately 50% who were aware). Similar awareness-to-active engagement statistics are also available at the national level, were one to consider that an estimated 36% of US adults are aware of physical activity recommendations, with only 10% meeting such benchmarks [18, 19]. There is evidence to suggest that increasing the density of community resources positively relates to physical activity levels [20], specifically with increasing overall exercise frequency [21]. Therefore, implementing such resources is a first step in promoting exercise in older adults, given the noted positive relationships between the presence of fitness resources within the community and physical activity levels [22]. Still, awareness of the benefits of exercise towards health is a critical determinant of exercise adoption and adherence [23]. Given that 50% of the current study population was aware of CBFR, it is plausible that many did not favorably view CBFR or were possibly not aware of specific opportunities within community centers to tailor physical activity and exercise to their preferences.

Provided the disconnect between awareness of CBFR and the use of these available resources, other factors pertaining to facility use are likely more influential. Barriers, both personal and environmental, represent factors that inhibit CBFR utilization. Among barriers measured in the current study across all spatial tiers, health and interest were the only significant predictors of not utilizing CBFR, although interest was the most commonly reported barrier. Health, in the context of reporting multiple chronic conditions, has been shown to decrease the amount of adults meeting physical activity guidelines by up to 30% [24]. In the current study, 34% reported multiple chronic conditions, suggesting that this sample population may have been in poor health overall, which resulted in low CBFR utilization rates. Although health status has been noted as a critical barrier [23], it has also been viewed as a powerful physical activity motivator among older adults [25]. Accordingly, this sample population also represents the massive potential benefit to promote health as a theme to increase interest in CBFR. Educational components have been shown to have positive relationships with physical activity and health [26]. Thus, intervening and educating on health is a logical first step towards bolstering interest rates, targeting both barriers reported for not utilizing CBFR.

Among other barriers, only transportation and health were significant barriers in the >1.6 to ≤3.2 km and >3.2 to 8.0 km radii, respectively. Approximately 10% of the sample population reported difficulties with transportation to CBFR. The term “transportation” is one of the most influential barriers to physical activity in older adults [27–29] and includes multiple contexts, spanning financial, health, distance, time, and built environment factors [30, 31]. Among factors related to transportation that require extended time and/or monetary investments and thus are less feasible to modify in the short term, are environmental aesthetics, safety, and walkability (sidewalks, traffic lights) [32]. Conversely, factors more easily modified are often specific to each individual. Given that both health and transportation were significant barriers in the current study, there is more evidence to suggest a poor level of health in the population that would inhibit transportation to CBFR and, therefore, result in low levels of utilization.

Based on current physical activity recommendations, the current sample population can be classified as, on average, sufficiently active with an average energy expenditure exceeding 1500 kcals/wk, assuming 100 kcals per 10 minutes of moderate intensity activity. Although this is higher compared to other reported activity levels in older adults, there were large variations in energy expenditure, from sedentary to extremely active. Only 132 participants (35.0%) reported actually engaging in over 1500 kcals/wk, providing evidence that bolsters the potential for CBFR to increase physical activity among those insufficiently active. In particular, such resources have been shown to be linked to increased participation in more intense, exercise-type behaviors [33], which is increasingly important, given the combination of low utilization rates of such resources and overall inactivity of the sample population (two-thirds not meeting recommended activity levels). Despite low utilization rates of CBFR, there was a marginal trend of increased utilization of exercise bikes, aerobic machines, and strength training equipment (in general) with increased distance from CBFR. Coupled with the observation that overall physical activity increased the further one resided from CBFR, this evidence reinforces the potential of CBFR to increase overall physical activity levels via exercise equipment should utilization rates increase.

This study is not without limitations. By design, the study was cross-sectional, so one is not able to glean causation between awareness and use of CBFR and overall physical activity levels. However, there is benefit to the random sampling of participants from surrounding neighborhoods, providing a large and diverse population to draw conclusions from. Another limitation is that physical activity data were obtained from subjective methodologies, specifically pertaining to the risk of participant bias based on expectant outcomes and recall error [34]. Considering such weaknesses, though, self-report questionnaires have been shown to accurately rank individuals across varying levels of physical activity [35].

## 5. Conclusions

Overall, closer proximity to CBFR did not impact awareness or utilization rates of such resources, while physical activity levels marginally increased the further one resided from CBFR. Future work in objectively assessing physical activity while utilizing CBFR is warranted to explore the utility of such resources to promote meaningful increases in energy expenditure in older adults, while investigating strategies to increase awareness and utilization of such resources.

## Figures and Tables

**Table 1:** Participant demographics (mean ± SD).

	All (*N* = 417)	≤1.6 km radius (*n* = 161)	>1.6 to ≤3.2 km radius (*n* = 114)	>3.2 to 8.0 km radius (*n* = 142)
Age (yrs, *n* = 414)	72.9 ± 7.7	73.4 ± 7.9	72.5 ± 7.7	72.6 ± 7.6
Height (m, *n* = 381)	1.7 ± 0.1	1.7 ± 0.1	1.7 ± 0.1	1.7 ± 0.1
Weight (kg, *n* = 385)	82.9 ± 20.0	83.9 ± 21.3	81.9 ± 19.5	82.6 ± 19.0
Body mass index (kg/m^2^, *n* = 373)	29.3 ± 6.6	29.7 ± 6.6	29.2 ± 6.0	29.0 ± 7.1
Gender (%, *n* = 414)	50.2	50.9	56.8	44.4
Ethnicity (%, *n* = 412)	82.3	81.3	80.2	87.9
Education (%, *n* = 409)	96.3	96.2	98.2	94.9
Income (%, *n* = 376)				
<$5,000	1.7	1.9	2.6	0.7
$5000–$14999	11.3	13.7	10.5	9.2
$15000–$24999	18.9	19.9	17.5	19.0
$25000–$34999	19.2	18.0	20.2	19.7
$35000–$49999	15.1	16.1	14.0	14.8
>$50000	24.0	19.9	24.6	28.2
Car (*n* = 417)	85.0	84.5	86.8	83.8

*Note*. Gender: percentage of female participants. Ethnicity: percentage of Caucasian participants. Education: percentage of those with at least a high school education. Car ownership reflects the percentage of participants that own a car.
